# The Causation of Dental Erosion

**Published:** 1894-12

**Authors:** A. P. Brubaker

**Affiliations:** Philadelphia


THE
### International Dental Journal.

Vol. XV. December, 1894. No. 12.

### Original Communications.[1]

### THE CAUSATION OF DENTAL EROSION.[2]

BY A. P. BRUBAKER, M.D., D.D.S., PHILADELPHIA.

Before proceeding to offer any facts or arguments relating to the causation or pathology of dental erosion, as it is observed on the labial surfaces of the incisor teeth, permit me to summarize the existing state of knowledge upon this subject. In the minds of most practitioners dental erosion is associated with the presence of an abnormal secretion, acid in character, discharged from some of the labial glands; the secretion bearing to the erosion the relation of cause and effect. This supposition is supported by the fact that if the inner surface of the lip be examined in cases of erosion, the orifices of a number of the labial glands will be found enlarged, the tissues around red and vascular, and upon pressure can be made to exude a thin watery fluid which is distinctly acid in character, as shown by its reaction with blue litmus-paper. It is further supposed that this acid, though secreted constantly, is by some observers regarded as inactive during the day, owing to its neutralization by the alkalies of the saliva, but that, at night in the absence of these alkalies, it exerts a decalcifying or disintegrating action upon the enamel.

[1] The editor and publishers are not responsible for the views of authors of papers published in this department, nor for any claim to novelty, or otherwise, that may be made by them. No papers will be received for this department that have appeared in any other journal published in the country.

[2] Read before the Odontological Society of Pennsylvania, October 13, 1894.

The lips are regarded as accessory agents, mechanically holding the secretion in contact with the teeth during the night and polishing them during the day. The tooth once decalcified falls an easy prey to the mechanical action of the tooth-brush. The various observations and experiments by which this knowledge has been evolved are the common property of the profession, and for which we are mainly indebted to Dr. James Truman[1] and Dr. E. C. Kirk.[2] In 1893, Dr. Safford G. Perry,[3] of New York, from personal experience and from the observations of patients, stated as his opinion that dental erosion is due to an excess of uric acid in the blood, and is to be therefore regarded as an evidence of the gouty diathesis.

According to Dr. Perry, systemic treatment will check the progress of the erosion, so that excavation and filling of the cavity is not always necessary. While the co-existence of these two pathological states is not to be denied, it is difficult to see how uric acid could give rise to a decalcification of the enamel unless it were excreted either by the salivary or mucous glands of the mouth. Dr. Perry, however, states that he has no proofs to offer that uric acid is present in the mouth or that it is this acid which causes the erosion, but he feels justified in asserting that that condition of the nutrition which predisposes to the production of uric acid gives rise to the particular acid of the labial secretion which does cause the erosion without stating what that acid may be.

The problem, therefore, which presents itself has several elements requiring solution. 1. What is the nature of this acid? 2. Is it capable of decalcifying enamel? 3. What is its origin?

The problem is a difficult one, and it is not with the idea of conveying the impression that the writer has solved it that he presents these observations to-night, but rather with the idea that lines of investigation may be suggested which others may pursue with success.

In the interpretation of any pathological condition, the primary essential is a clear conception of the anatomy and physiology of the structures involved, without which no progress can be made. Permit me, therefore, to direct your attention to the glands of the mouth in general and to the glands of the lips in particular. As is well-known, the mouth is abundantly supplied with glands which, in recent years, have received very considerable attention; they

---

[1] International Dental Journal, vol. xiv., No. 4.

[2] Dental Cosmos, vol. xxix. pp. 55, 56.

[3] International Dental Journal, vol. xiv., No. 4.

have been divided by Professor Heidenhain into two classes, which differ in the chemical composition by their secretion as well as in the microscopical structure of their secreting elements.

The glands of the first class or type secrete a thin watery fluid containing albumen, salts, and a ferment body. They have, in consequence, been termed albuminous glands. To this class belong the parotid, a portion of the submaxillary, and a portion of the glands found in the tongue and in the nose.

The glands of the second class or type secrete a more or less viscid fluid which contains but a small quantity of albumen and salts, but a relatively large quantity of an albuminoid substance termed mucin. These glands have therefore been termed mucous glands. To this class belong a portion of the submaxillary gland, the sublingual, a portion of the glands of the tongue, the glands of the cheeks, palate, pharynx, and lips.

The topographical distribution of the two types of glands has been very carefully studied in man and different classes of animals. According to Podwisotski, the albuminous glands preponderate very markedly in sheep, goats, and polecats; less so in the pig, horse, guinea-pig, fox, dog, hedgehog, and squirrel.

The mucous glands, on the contrary, preponderate considerably in the bat; less so in the armadillo, mole, and cat. Both types of glands are found in equal proportions in men, monkeys, and rats.

For the purpose in view, I will confine my remarks to the labial glands, merely alluding to the fact that all mucous glands in their structure are in all essential respects similar.

If the upper lip be removed from the body of an individual recently dead, and the skin and orbicular muscle be dissected off, the labial glands will come prominently into view. They are found most abundantly in the upper lip. Though arranged quite irregularly, they are more numerous towards the centre, less so at the extremities of the lip. The body of the gland is rounded, about the size of a small shot, and provided with a narrow duct which is sometimes twisted. These glands are held in position by connective tissue, furnished by blood-vessels and supplied with nerves.

A microscopical section of a labial gland shows that, as the main duct passes through the mucous membrane into the submucous tissue, it branches into two or more ducts, which terminate in enlarged alveoli. The wall of the duct is lined by low, granular, nucleated epithelium, which stains readily with carmine. In the beginning of the secretory part of the gland the epithelial cells are changed in character, becoming enlarged, columnar, glassy in appearance, and, as shown by their less readily staining with carmine, changed in chemical composition.

The function of the mucous gland generally is to secrete a mucous fluid which aids in mastication and deglutition, while that of the labial gland is to lubricate the surfaces of the teeth so as to permit of free and easy movements of the lips in articulation.

The chemical composition of the secretion of the mucous gland was determined some years ago by Bidder and Schmidt. According to these observers this fluid contains water, mucin, and inorganic salts, the chief of which is sodium phosphate, which imparts to the fluid its alkalinity.

Without going into unnecessary details, permit me to present a summary of the process of secretion, as it has been shown to take place in mucous glands in general. The raw material out of which the gland-cells elaborate the characteristic secretion is furnished by the blood-vessels surrounding the gland. As the blood flows through the capillary vessels the plasma passes through the capillary wall into a surrounding space known as the lymph-space. From this space the epithelial cells absorb those specific materials, which are transformed within the cells into mucin. The water and inorganic salts undergo no change apparently. According to the quantity of the blood furnished will be the supply of raw material. Hence during the periods of glandular activity the blood-vessels are enlarged, this enlargement being brought about by the action of vaso-motor nerves. Coincident with this increased blood-supply the gland-cells themselves are stimulated to increased activity by a special class of nerves, which may be termed secretory. As a result of both influences increased secretion immediately takes place. The nerve impulses exciting both blood-vessels and nerves originate in special nerve-centres in the medulla oblongata. These centres, however, may be thrown into activity in two ways. First, from irritation at the periphery and transmitted through sensory nerves; second, by the character of the blood plasma which flows around the centres. With the cessation of the irritation the centres cease to act and the phenomena of secretion subside.

Here, as elsewhere, however, the pathological law holds good, that the existence of a continuous irritation causes permanent dilatation of blood-vessels and hyperæmia, impaired activity of the epithelial cell, and the production of an altered secretion. In the light of this statement, what does the labial gland present for our inspection? Certainly a change from a normal to an abnormal, from a physiological to a pathological state, as shown by the persistent hyperæmia, a secretion altered in viscidity, and changed from an alkaline to an acid reaction.

The questions which now arise are: 1. What is the nature of the acid here present? 2. Is it capable of decalcifying the enamel? 3. What is its origin?

As stated in the beginning of this paper, from the co-existence of dental erosion and gouty manifestations, an impression has become widespread that the acid in question is uric acid, and necessarily an excretion from the blood by the mucous glands. To this interpretation I must express my complete dissent, and for the following reasons:

In the first place, it would be a most unusual occurrence physiologically for a mucous gland to take upon itself the excretion of uric acid when the natural excretory organs, the kidneys, are in fair condition. In the second place, uric acid is soluble even in hot water only to the extent of one grain in eighteen hundred,—in other words, to the extent of one grain in four ounces of water. As the quantity of fluid secreted by a few diseased labial glands only amounts to but a few drops in comparison, the amount of uric acid must necessarily be small and insufficient to produce any destructive influence on the enamel. In the third place, uric acid is not, as its name implies, always an acid body. It is considered by chemists as an amphoteric body,—that is, one which is as frequently acid as alkaline, and often neutral. In the fourth place, uric acid is incapable both from its small amount and feeble combining power of decalcifying enamel.

In answer to the first question, it may be said that the acid which is theoretically most likely to be present in the secretion is one derived from its normal constituents. It will be recalled that among the salts there is present in considerable quantity sodium phosphate, a distinctly alkaline salt. One of the peculiarities of this salt is that it readily parts with one atom of its sodium when brought into relation with carbonic acid, when it becomes the well-known acid sodic phosphate.[1] The source of the $CO_2$ must be sought for in the metabolism of the gland-cells. In the normal activity of the glands the $CO_2$ produced appears to be insufficient

---

[1] Since writing this paper, my attention has been called to an editorial in the *Dental Cosmos*, June, 1894, by Dr. E. C. Kirk, on "The Oral Manifestations of Lithæmia." At the close of this editorial the writer states that the deposition of uric acid in or around the labial gland "might by its irritant action bring about an alteration of function of the gland which would cause it to secrete an acid mucus, the acidity of which would be due to some other substance than uric acid,—the acid sodium phosphate, for example."

in amount to bring about this conversion. But with increased vascularity and, in consequence, heightened cell activity, the production of $CO_2$ must be very considerably increased. Under such circumstances it is easy to see that it might combine with all the sodium phosphate with the production of the acid salt. The reaction would be as follows:

$$Na_2HPO_4 + H_2CO_3 = NaH_2PO_4 + NaHCO_3.$$

The presence of this acid, it must be confessed, has not been demonstrated, inasmuch as there are no known tests by which it can be detected when dealing with such small amounts. In looking at the list of organic acids which are capable of decalcifying enamel, this is the only one which apparently could come from a mucous gland.

Assuming for the moment that the acid present in the secretion is the acid sodic phosphate, the next question is, Can it decalcify enamel? The possibility of such a capacity is apparent from an examination of the following changes:

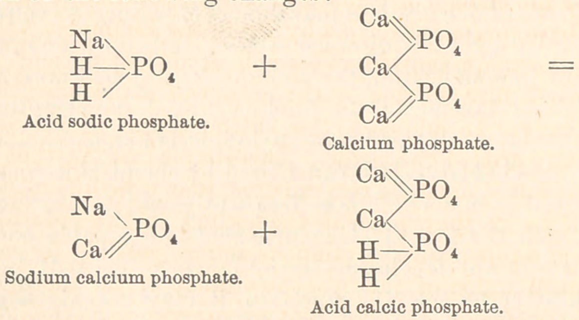

When a molecule of the acid sodic phosphate is brought into relation with a molecule of calcium phosphate, a double decomposition takes place. On the one hand, one atom of the calcium displaces the two atoms of hydrogen, thus forming a compound known as sodium calcium phosphate,—an insoluble body. On the other hand, the two displaced atoms of hydrogen replace the calcium atoms in the calcium phosphate, thus forming in the enamel the acid calcic phosphate, which is more soluble than the original calcium phosphate. With the appearance now of a new molecule of the acid sodic phosphate from the gland, a further decomposition takes place as follows:

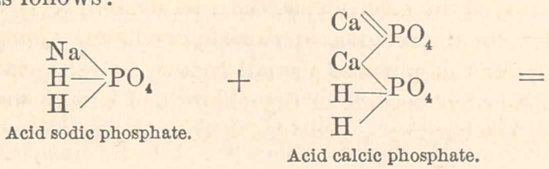

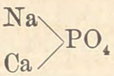

Sodium calcium phosphate.

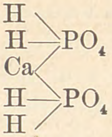

Diacid calcic phosphate.

Once again the calcium atom of the newly-formed acid calcic phosphate is replaced by the hydrogen atoms, with the formation of the old superphosphate or the diacid calcic phosphate, which is very soluble. While the calcium atom combines with sodium and phosphoric acid atoms, and forms the sodium-calcium phosphate.

In this way it becomes conceivable how one by one the molecules of the calcium phosphate of the tooth are gradually disintegrated by being converted first into the mono and secondly into the diacid calcic phosphate.

To convert this theoretical possibility into an actuality I immersed the enamel of a tooth in a weak solution of the acid sodium phosphate, prepared for me by Professor Leffman. At the end of a week the tooth showed two small eroded spots and a number of transverse furrows due to the action of a tooth-brush which was used daily. In addition, the cutting-edges of the tooth show a distinctly eroded character. This simple test may not be very conclusive, but it must be remembered that it is difficult to realize the conditions as they present themselves in the mouth,—namely, a uniform temperature, continuous action, possibly for many months, continued action of the lips, the vitality of the tooth, etc.

In conclusion, I would like to suggest a method of treatment which might be of some value. Even admitting that the underlying cause is the presence of uric acid in the blood, and that the discharge of acid and the subsequent erosion can be arrested by an anti-gout treatment, it is, nevertheless, well known that with a return of the gouty condition there will be a re-establishment of the gland disturbance. Now if the gland, which is the immediate cause of the acid secretion, were removed, even though the gouty phenomena returned, there could be no further discharge of the secretion.

It has occurred to me that the diseased gland can be removed or destroyed by electrolysis, the method employed by the electro-surgeon for the removal of superfluous hairs. These practitioners employ for this purpose a small battery of cells, either the fluid bichromate of potassium or dry chloride of silver cells. The *negative* pole of the battery is provided with a fine platinum wire, which is carefully inserted into the hair-follicle. The *positive* pole is provided with a sponge electrode, which is placed in a small bowl of water. When the needle is carefully adjusted, the patient inserts two fingers in the water. After the lapse of a minute or two a white froth makes its appearance around the mouth of the follicle, which is an indication that the gland-cells have undergone decomposition. The hair can then be readily extracted. After this destruction of the follicle no hair will ever grow.

Now, if this same method were applied to the labial gland in the same way, their decomposition could be brought about with a total cessation in the discharge of any secretion. The lips could be easily everted, and by employing a bent electrode the orifice of the gland could be easily penetrated.

